# CRISPR Recognition Tool (CRT): a tool for automatic detection of clustered regularly interspaced palindromic repeats

**DOI:** 10.1186/1471-2105-8-209

**Published:** 2007-06-18

**Authors:** Charles Bland, Teresa L Ramsey, Fareedah Sabree, Micheal Lowe, Kyndall Brown, Nikos C Kyrpides, Philip Hugenholtz

**Affiliations:** 1 Department of Computer Science, Jackson State University, Jackson, MS 39217, USA; 2 DOE Joint Genome Institute, Walnut Creek, CA 94598, USA; 3 Department of Computer Science and Engineering, University of Nebraska-Lincoln, Lincoln, NE 68504, USA

## Abstract

**Background:**

Clustered Regularly Interspaced Palindromic Repeats (CRISPRs) are a novel type of direct repeat found in a wide range of bacteria and archaea. CRISPRs are beginning to attract attention because of their proposed mechanism; that is, defending their hosts against invading extrachromosomal elements such as viruses. Existing repeat detection tools do a poor job of identifying CRISPRs due to the presence of unique spacer sequences separating the repeats. In this study, a new tool, CRT, is introduced that rapidly and accurately identifies CRISPRs in large DNA strings, such as genomes and metagenomes.

**Results:**

CRT was compared to CRISPR detection tools, Patscan and Pilercr. In terms of correctness, CRT was shown to be very reliable, demonstrating significant improvements over Patscan for measures precision, recall and quality. When compared to Pilercr, CRT showed improved performance for recall and quality. In terms of speed, CRT proved to be a huge improvement over Patscan. Both CRT and Pilercr were comparable in speed, however CRT was faster for genomes containing large numbers of repeats.

**Conclusion:**

In this paper a new tool was introduced for the automatic detection of CRISPR elements. This tool, CRT, showed some important improvements over current techniques for CRISPR identification. CRT's approach to detecting repetitive sequences is straightforward. It uses a simple sequential scan of a DNA sequence and detects repeats directly without any major conversion or preprocessing of the input. This leads to a program that is easy to describe and understand; yet it is very accurate, fast and memory efficient, being O(*n*) in space and O(*nm*/*l*) in time.

## Background

Repetitive sequences are abundant in bacteria and archaea, accounting for close to 5% of the genome size in many organisms [1,2]. These repetitive sequences come in various forms/sizes and may be found dispersed throughout a genome, clustered in close proximity or arranged contiguously. The identification of repeats has proven to be of significance, as they provide insight into the functional and evolutionary roles of various organisms [3-7].

This study centers on a recently recognized family of repeats known as Clustered Regularly Interspaced Palindromic Repeats (CRISPRs). Since their description by Mojica *et al*. [8], CRISPRs have attracted a great deal of interest [9-16]. CRISPRs have been found only in the genomes of prokaryotes and are composed of short direct repeats currently known to range in sizes from 21 – 47 base pairs. This family of repeats is unique in that they are interspaced by non-repeating sequences of similar size. CRISPRs were found in approximately 40% of bacterial genomes investigated [14]. Of those genomes with CRISPRs present, about one half contained multiple CRISPR loci. The average number of repeats per loci was found to be 27, with an average repeat length of 32 base pairs. Although knowledge of the characteristics of CRISPRs continues to grow, their complete function is still not yet known. One recently verified hypothesis, however, is that they defend against invading viruses [16].

Several software applications are available for identifying various forms of repeats. However, because the focus on CRISPR elements is recent, only one CRISPR-specific tool has been published for their automatic detection [17]. Identification based on generic repeat searching applications such as Patscan [18] require considerable manual post-processing. In this study, a new tool for the automatic detection of CRISPR elements is presented. This software program, CRISPR Recognition Tool (CRT), uses a simple sequential search technique that detects repeats directly from a DNA sequence. Unlike most repeat detection techniques, the algorithm presented in this paper does not rely on the use of the suffix tree or alignment matrix as a central data structure. Instead, repeats are discovered directly from the DNA. As a result, this technique is very efficient in terms of memory usage, and it is much easier to understand and implement than most other methods. Despite its simplicity, the presented algorithm is able to achieved impressive execution speed when compared to other repeat detection tools.

## Implementation

CRT's search for CRISPRs is based on finding a series of short exact repeats of length *k *that are separated by a similar distance and then extending these exact *k*-mer matches to the actual repeat length. The value of *k *should be small and less than the length of the shortest repeat to be detected. By making *k *small, string comparison is faster and the likelihood of finding exact matches between approximate repeats is increased. Once actual repeats are found, they are filtered to remove those that do not meet CRISPR specific requirements.

### Searching for exact *k*-mer matches

The algorithm begins its search for repeats with a left-to-right scan of a sequence using a small sliding *search window *of length *k*. The value in the search window represents a candidate repeat, and each time the window reads a new *k*-mer, the algorithm searches forward for exact *k*-mer matches. When searching for each successive match, the search space can be restricted to a small range, called *search range*. Given a *k*-mer that begins at position *i*, any exact *k*-mer match, if one exists, should occur in the range:

[*i *+ *minR *+ *minS *.. *i *+ *maxR *+ *maxS *+ *k*]

**Figure 1:**
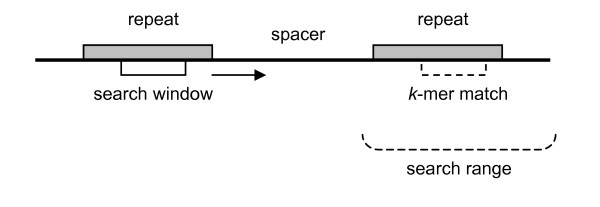
**An occurrence of a CRISPR**. Repetitive sequences are detected by reading a small search window and then scanning ahead for exact *k*-mer matches separated by a similar distance.

Here, *minR *and *maxR *refer to the lengths of the smallest and largest repeats to be detected. The lengths of spacers, which are the similarly sized non-repeating regions between repeats, are referred to by *minS *and *maxS *(See Figure 1). Since CRISPRs are to some degree evenly spaced, the distance between the initial repeats can be used to approximate the spacing between subsequent exact *k*-mer matches. Thus the size of the search range can be reduced further, resulting in faster processing time.

The size of the search range has a direct effect on the processing time of the algorithm, with smaller ranges being more desirable. Thus, the algorithm runs fastest when there is little variation between the sizes of the smallest/largest repeats and the smallest/largest spacers.

### If exact *k*-mer matches are found

The search described above detects a succession of similarly spaced repeats of length *k*. Since these repeats do not represent the true length of the repeating pattern, they must be extended (left and right) to the actual repeat length. Any method for extending repeats must consider that mutations occur in DNA sequences, so, repeats may not be exact. The approach taken is this paper is to read the characters to the left or right of all repeats and compute occurrence percentages for each base, ACGT. If there is a character that has an occurrence percentage greater than or equal to some preset value, *p*, the repeats are extended. For example, if extending left, a *p *value of 100% extends exact *k*-mer matches to exact (*k*+1)-mer matches only if the character to the left of all repeats within the CRISPR is the same. Thus, for *p *= 100%, exact repeats are detected, while lower values allow for the detection of approximate repeats. This method of extending repeats works well for CRISPRs, give an appropriate value for *p *(CRT uses a default value of 75%).

### If no exact *k*-mer matches are found

If no exact *k*-mer matches are found, the search window advances forward and the process described above is repeated. The search window can actually advance forward in intervals greater than one without missing any repeats. The size of this interval is one of the major factors contributing to the speed of the presented algorithm.

The key to being able to advance at greater intervals is guaranteeing that the search window will never skip any repetitive sequence during its traversal of the DNA sequence. That is, the interval at which the search window advances must be small enough that the entire window will (at some point) fall entirely within each repeat. The length of this interval is dependent on the size of the search window, *k*, and the length of the smallest repeats to be identified, *minR*. It can be computed as follows.

*interval *= max {*minR *- (2*k *- 1), 1}

Longer repeats produce larger intervals, as do smaller search windows. Larger intervals result in significant improvements in speed because less data is analyzed. For example, for *minR *= 21 and *k *= 6, the search window can skip 10 positions each time it advances. Thus, processing a DNA sequence of length 1,000,000, for the most part, becomes equivalent to processing a sequence of length 1,000,000/10 (or 100,000).

Although smaller search windows improve processing speed, if continuing to reduce their size, the speed of the algorithm may at some point worsen. This is because smaller search windows increase the likelihood of the program finding short repetitive sequences that are not really part of a true CRISPR element, but happen by chance. This will cause the program to spend more time processing repeats that are actually false positives. As an example, for a search window of length *k *= 3, there is a 1/4^3 ^chance that any 3-mer will be a match to the search window. This assumes that all four bases are equally likely to appear at any position.

### Filtering

Many of the candidate CRISPRs found from the process described above will either be contiguous repeats or repeats with incorrect starting and/or ending positions. To remove unwanted repetitive sequences, filters are applied. The first filter checks that the candidate CRISPR is composed of short repeats (between *minR *and *maxR *in length). If that condition is met, the spacers are checked for being non-repeating and similarly sized. Filtering is fast because most repetitive sequences do not make it deep into the process. Also, when testing for similarly sized/non-repeating spacers, it is only necessary to check the first few spacers of the CRISPR.

The final part of program checks the left and right flanks of a CRISPR in case repeats were missed because of too many mismatches. The flank check is less strict than the initial search for repeats in that it does not look for short exact matches. Instead, the discovered repeats within the CRISPR are used for comparison (using hamming distance) to detect any nearby approximate repeats. The flank check is important for two reasons. The likelihood of missing repeats with mismatches increases when advancing the search window in intervals. Furthermore, according to Jansen *et al*. [10], the last or last few repeats of CRISPRs contain mutations in most organisms, and about one-third of CRISPRs have the last repeat truncated.

### Time and Space

The CRT algorithm moves a *search window *through a sequence in intervals, at each step scanning the *search range *for the pattern in the search window. Searching for a pattern in a text can be done using any fast search algorithm. The Boyer-Moore [19] string-matching algorithm is used here. It is linear in time (on average, the algorithm has a sublinear behavior). Thus, the running time of the algorithm for finding CRISPRs as described in this paper is O(*nm*/*l*), where *n *is the length of the DNA sequence, *m *is the length of the search range and *l *is the interval at which the search window advances. (The actual behavior of the algorithm is linear and is supported by empirical evidence in the following section.) The algorithm is also linear in space, since repeats are detected directly from the input sequence with no additional major structures required.

## Results

CRT (version 1.0), Pilercr (version 1.0) [17] and Patscan [18] were compared based on execution speed and ability to correctly identify CRISPRs. Patscan is a generic pattern discovery application that identifies repetitive sequences given a user-specified input pattern. The number of repeats that Patscan detects must be predefined, and the tool has no mechanism for distinguishing repeating and non-repeating regions of CRISPRs. Thus, considerable manual processing of the output is required in order to remove unwanted results and to extend repetitive sequences beyond the fixed size limit set by the input pattern. Pilercr is a recently developed tool designed specifically for the automatic detection of CRISPR elements. It is based on the Piler [20] program, which utilizes alignment matrices for detecting contiguous repeats.

Both Patscan and Pilercr were implemented in the C programming language. CRT was developed using Java. All tools were tested on finished microbial genomes available in the IMG version 1.5 database [21]. Each was run under Cygwin version 1.5.21 on a PC having the following specifications: Windows XP operation system, Pentium 3.4 GHz processor, 1.0 GB RAM.

### Speed Evaluation

**Figure 2:**
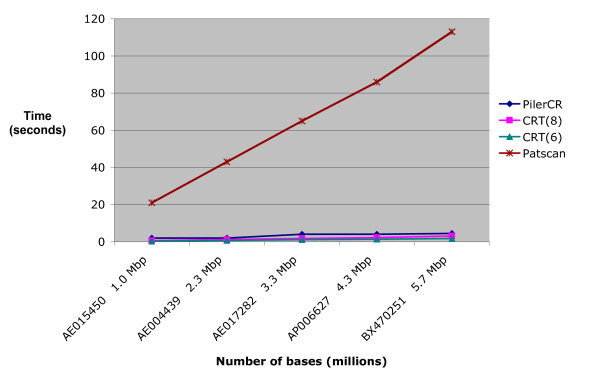
**Running time based on genome size, using repeat length 21–37 and spacer length 19–48**. Running times for the three compared search tools, based on genome size (CRT is listed twice, once for windows size 6 and once for window size 8). The y-axis represents time in seconds. The x-axis lists the genome accession numbers, followed by their sizes in million base pairs (Mbp). As the size of the genomes increase, it can be seen that running times of the search tools increase at different rates. Below, the corresponding organism names are given. [IMG:AE015450] Mycoplasma gallisepticum (strain R(low)) [IMG:AE004439] Pasteurella multocida (strain Pm70) [IMG:AE017282] Methylococcus capsulatus (strain Bath/NCIMB 11132) [IMG:AP006627] Bacillus clausii (strain KSM-K16) [IMG:BX470251] Photorhabdus luminescens (subsp. laumondii, strain TT01).

**Figure 3:**
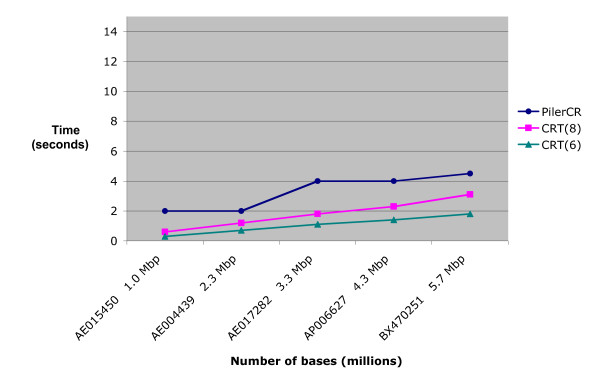
**Running time based on genome size, excluding Patscan**. Running times for the search tools, excluding Patscan. The parameter values and organisms are the same as that in Figure 2. However, by removing Patscan, a better comparison of the execution speeds of PilerCR and CRT can be achieved.

**Figure 4:**
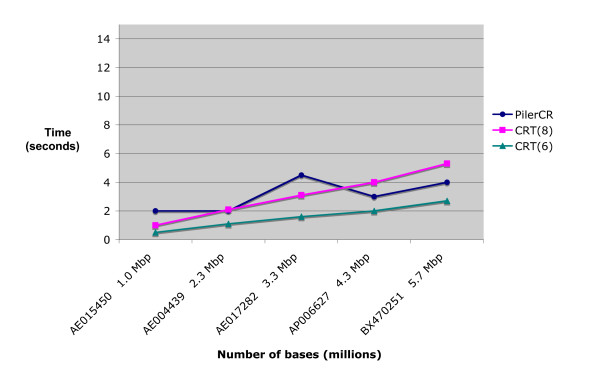
**Running time based on genome size, using repeat length 19–50 and spacer length is 19–60**. Running times for two of the compared search tools, based on genome size (CRT is listed twice, once for windows size 6 and once for window size 8). This figure is the same as Figure 3, except the ranges of the repeat length and spacer length to be detected are increased.

Figures 2, 3 and 4 show the execution times of the three tools. The x-axis contains the accession number for the analyzed organisms followed by their approximate number of base pairs in millions (Mbp). As the number of repeats may affect execution time, only genomes with similar repeats counts were used (between 70 and 80). Figures 2 and 3 are based on a search with repeat size 21 – 37, spacer size 19 – 48, and minimum number of CRISPR repeats 3. CRT required an additional setting for search window length. It was tested for values 6 and 8. Figure 4 shows results when searching for longer repeats of size 19 – 50 and spacer size 19 – 60 (this is beyond the range of any CRISPRs found in any previous work). Patscan is not included in this figure, or any subsequent figures, because it's extended running times flattens the other graph lines, making it difficult to compare the other tools (see Figure 2).

The speed of CRT and Pilercr is very impressive and a huge improvement over the previous technique of CRISPR detection using Patscan, as shown in Figures 2 and 3. CRT achieves the best performance, being able to process a DNA sequence of nearly 6 million bases in about 3 seconds using a search window of size 8, CRT(8), and in about 2 seconds for a search window of size 6, CRT(6).

Figure 4 shows a slight decrease in the performance of CRT as the range in the size of repeats to be detected is increased (see the previous section). The performance of Pilercr, however, appears to be independent of the size range of repeats. For these settings, the speed of CRT(8) and Pilercr are about the same, with CRT(6) performing best.

**Figure 5:**
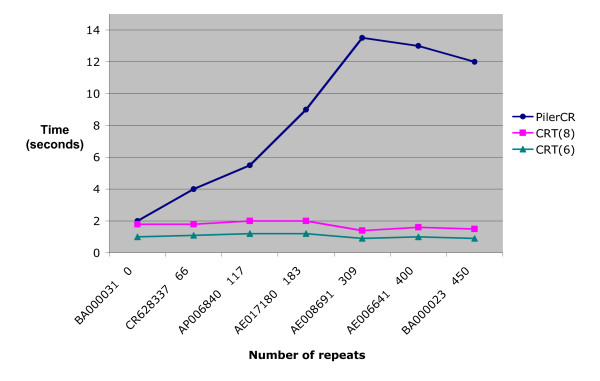
**Running time based on number of repeats, using repeat length 21–37 and spacer length 19–48**. Running times for two of the compared search tools based on number of repeats processed. CRT is listed twice, once for windows size 6 and once for window size 8. The y-axis represents time in seconds. The x-axis lists the genome accession numbers, followed by the number of repeats detected in the genome. As the size of the genomes increase, it can bee seen that running times of the search tools increase at different rates. Below, the corresponding organism names and the number of CRISPR loci are given. All genomes are close in size (2.7 – 3.8 Mbp). [IMG:BA000031] Vibrio parahaemolyticus (serovar O3:K6, strain RIMD 2210633) loci: 0 [IMG:CR628337] Legionella pneumophila (strain Lens) loci: 2 [IMG:AP006840] Symbiobacterium thermophilum (strain IAM 14863/T) loci: 3 [IMG:AE017180] Geobacter sulfurreducens (strain ATCC 51573/PCA) loci: 2 [IMG:AE008691] Thermoanaerobacter tengcongensis (strain MB4/JCM 11007) loci: 3 [IMG:AE006641] Sulfolobus solfataricus P2 loci: 7 [IMG:BA000023] Sulfolobus tokodaii str. 7 DNA loci: 7.

In the previous example, execution speed was analyzed based on increasing genome size. In Figure 5, speed is analyzed for increasing number of repeats. Only genomes of similar sizes were used (2.7 – 3.8 Mbp). The repeat size is 21 – 37 and spacer size is 19 – 48. The figures show that CRT performs better than Pilercr for larger number of repeats. Like Pilercr, whose speed appears to be independent of the size range of repeats, CRT's speed is independent of the number of repeats contained in a genome. Actually, CRT improves slightly in processing time as the number of repeats increases. This is because it is able to process sections of a sequence containing repeats very fast, as explained in the previous section.

### Retrieval Evaluation

In order to assist in determining the effectiveness of the three tools in identifying CRISPR elements, three evaluation measures were used: quality, precision and recall.

### Quality

Detected CRISPRs are sometimes inconsistent with their actual form in a sequence. This generally results because DNA repeats are not always exact, and consequently are often difficult to correctly identify. Three common types of inconsistencies were identified in this study. **Type I **inconsistencies occur when a tool reports a CRISPR that is incomplete (that is, the CRISPR does not contain all of the repetitive sequences). **Type II **inconsistencies occur when repeats within a CRISPR do not begin and/or end at the correct position. For example, A CRISPR that actually begins with the sequence GTTTAC may be reported as beginning with TTTAC. In this case, it can be seen that the reporting tool is off by one position. **Type III **inconsistencies occur when a CRISPR is split. For example, a single CRISPR containing 10 repeats may be reported as two CRISPRS, each containing 5 repeats.

Let quality represent the likelihood that a CRISPR reported by a search tool does not contain an inconsistency of Type I, Type II or Type III. Based on this definition, there is no distinction between a CRISPR with one inconsistency and a CRISPR with three inconsistencies. Given the set of CRISPRs resulting from a search, let *a *be the total number of CRISPRs reported and *b *be the total number of CRISPRs containing at least one inconsistency. Assuming *a *> 0, quality (*q*) for a search tool can be computed as follows.

q=1−ba
 MathType@MTEF@5@5@+=feaafiart1ev1aaatCvAUfKttLearuWrP9MDH5MBPbIqV92AaeXatLxBI9gBamXvP5wqSXMqHnxAJn0BKvguHDwzZbqegyvzYrwyUfgarqqtubsr4rNCHbGeaGqiA8vkIkVAFgIELiFeLkFeLk=iY=Hhbbf9v8qqaqFr0xc9pk0xbba9q8WqFfeaY=biLkVcLq=JHqVepeea0=as0db9vqpepesP0xe9Fve9Fve9GapdbaqaaeGacaGaaiaabeqaamqadiabaaGcbaGaemyCaeNaeyypa0JaeGymaeJaeyOeI0YaaSaaaeaacqWGIbGyaeaacqWGHbqyaaaaaa@43E1@

### Precision and Recall

Quality alone is insufficient for measuring performance, as it does not consider the cost of failing to retrieve relevant CRISPRs or the cost of mistakenly retrieving instances that are not CRISPRs. For evaluating inconsistencies of these types, precision and recall are used.

Precision and recall are measures commonly used in the field of information retrieval (IR) when evaluating search algorithms. Their definitions are based on true positives, false positives and false negatives. The descriptions given here are expressed in terms of the tools evaluated in this study.

True positive (TP): the number of instances retrieved that were CRISPRs, False positive (FP): the number of instances retrieved that were not CRISPRs, False negative (FN): the number of instances not retrieved that were CRISPRs.

Determining FN can be problematic because it requires the total number of CRISPRs in the dataset to be known. As is often done in IR, in this study FN is estimated using the composite result sets from all of the available searches tools.

Using the definitions above, precision (*p*) and recall (*r*) can be computed as follows.

p=TPTP+FPr=TPTP+FN
 MathType@MTEF@5@5@+=feaafiart1ev1aaatCvAUfKttLearuWrP9MDH5MBPbIqV92AaeXatLxBI9gBaebbnrfifHhDYfgasaacH8akY=wiFfYdH8Gipec8Eeeu0xXdbba9frFj0=OqFfea0dXdd9vqai=hGuQ8kuc9pgc9s8qqaq=dirpe0xb9q8qiLsFr0=vr0=vr0dc8meaabaqaciaacaGaaeqabaqabeGadaaakeaafaqabeqacaaabaGaemiCaaNaeyypa0ZaaSaaaeaacqWGubavcqWGqbauaeaacqWGubavcqWGqbaucqGHRaWkcqWGgbGrcqWGqbauaaaabaGaemOCaiNaeyypa0ZaaSaaaeaacqWGubavcqWGqbauaeaacqWGubavcqWGqbaucqGHRaWkcqWGgbGrcqWGobGtaaaaaaaa@415F@

Precision is the ratio of the number of instances correctly identified to all the instances retrieved. Given an instance from the result set, it represents the likelihood of that instance being a CRISPR. Thus, precision can be used to answer the question, "Did the retrieval system identify a lot of junk (or instances that were not CRISPRs)?"

Recall is the ratio of the number of instances correctly identified to the total number of instances that are CRISPRs (whether retrieved or missed). Thus, recall can be used to answer the question, "Were all of the CRISPRs retrieved?"

In [14], Godde and Bickerton documented CRISPRs in 101 species with the use of Patscan. From that set, a random sample of size 27 was selected for comparison with results from CRT and Pilercr (using default parameter settings). Between Patscan, CRT and Pilercr, a total of 83 distinct CRISPRs were identified. Using the collective information, quality, precision and recall were computed for each tool. The results are presented in Table 1 under the heading CRISPRs with Cas genes. Note that precision is not applicable for Patscan, because false positives are removed during manual post-processing. Also, the results for CRT are based on a search window length of 8. A search window length of 6 would produce similar precision/recall results, but would have a slightly lower quality score, because the likelihood of Type III inaccuracies is slightly increased.

**Table 1:** Performance evaluation measures for the examined tools.

	CRISPRs with Cas genes			CRISPRs with/without Cas genes		
	
	Quality	Precision	Recall	Quality	Precision	Recall
CRT	.95	.99	.99	.90	.89	1
Pilercr	.77	1	.95	.75	1	.86
Patscan	.74	n/a	.89	--	--	--

A comparison of the three search tools, based on measures quality, precision and recall. The higher scores for CRT and Pilercr show that automatic detection of CRISPRs can be very reliable, even more so than with the use manual post-processing as is done with Patscan. The results in the left half of the table are for CRISPRs containing Cas genes. Because the authors suspect that CRISPRs with Cas genes have fewer mutations, and are thus easier to detect, a second experiment was performed using randomly selected finished genomes. The results of this second experiment are shown in the right half of the table. As expected, slightly lower scores resulted, and they should better reflect the effectiveness of the tools.

The high scores for CRT and Pilercr show that automatic detection of CRISPRs can be very reliable, even more so than with the use of manual post-processing as is done with Patscan. However, it is not clear whether the lower scores for Patscan were mostly from the human involvement in the detection process or from the Patscan algorithm.

The quality score was highest for CRT. The lower score for Patscan was due entirely to Type I inconsistencies. The categories of inconsistencies for Pilercr were evenly spread, with Type I and Type II inconsistencies usually missing by only small amounts. Precision was highest with Pilercr, while CRT had the best recall score. In this application of precision/recall, recall is more significant as it gives an indication of the number of CRISPRs that were missed by a search tool. Although precision is important, a more sensitive tool that detects most CRISPRs but also reports a few repetitive sequences that are not really CRISPRs is more desirable than a less sensitive tool that misses several CRISPRs but reports very few false positives.

As mentioned above, in order to include Patscan in retrieval evaluations, results were used from Godde and Bickerton. However, they reported CRISPRs only for species that had CRISPR-associated (Cas) genes [10]. The authors of this study suspect that CRISPRs with Cas genes may have fewer mutations, thus they are easier for search tools to detect. As a result, the tools have higher evaluation scores. For this reason, a second experiment was undertaken using 80 randomly selected finished genomes from the IMG version 1.5 database. Using CRT and Pilercr, a total of 51 distinct CRISPR elements were identified within the 80 genomes. The evaluation scores are shown in Table 1 under the heading CRISPRs with/without Cas genes. These results should be more reflective of the performance of the tools for a typical search. Almost all measures show a reduction in performance. The most noticeable difference is a decrease in precision for CRT and a decrease in recall for Pilercr.

## Discussion

The importance of identifying repetitive sequences is clear; however, the considerable size of many genomes makes fast and efficient repeat detection very challenging. Consequently, many detection techniques convert sequences to an alternative representation in an attempt to make analysis more efficient. A frequently used representation is the suffix tree [22]. Here, a DNA sequence is converted into a tree structure containing indices to all suffixes in the original sequence. By traversing the tree, an algorithm is able to find all occurrences of any pattern in time proportional to the size of the pattern. Because of the impressive speed of suffix trees, they have been widely used in DNA repeat detection [23-26]. The increased speed, however, comes at a cost. First, even before the search for repeats can begin, the suffix tree must be constructed from the sequence data. Second, after it is constructed, the tree can consume large amounts of memory.

Another technique frequently used for detecting repeats involves computing alignment matrices from DNA sequences [27,28]. Once implemented, the matrix can be used to find repeated regions in the sequence using one of several algorithms [22,29-31]. These algorithms, however, can be problematic because of extended processing times.

Unlike most repeat detection techniques, the algorithm presented in this paper does not rely on the use of the suffix tree or alignment matrix as a central data structure. No major conversion or preprocessing of the input is required. Instead, repeats are discovered directly from the DNA sequence using a simple left-to-right skip search technique with localized iterative extensions of identified repeat arrays in order to find exact boundaries. As a result, CRT is very efficient in terms of memory usage, at O(*n*), and O(*nm*/*l*) in time. Thus, a standard desktop machine is sufficient for processing large prokaryotic genomes, usually in a matter of seconds.

Future research plans are to modify the presented algorithm so that it is also able to identify contiguous repeats. Because of the nature of the CRT algorithm, the tool would not be practical for detecting very short patterns of sizes 2 – 4 nucleotides, for example. CRT is fastest when identifying longer repeats, and when there is little variation between the sizes of the smallest and largest repeats to be detected. Also, the tool is fast when processing genomes with large numbers of repeats; so, CRT may be useful for detecting contiguous repeats in eukaryotes, which tend to have more repetitive sequences than prokaryotic genomics.

## Conclusion

In this paper a new tool was introduced for the automatic detection of CRISPR elements. This tool, CRT, was shown to be a significant improvement over the current technique for CRISPR identification using Patscan. CRT's approach detects repeats directly from a DNA sequence. This leads to a program that is easy to describe and understand, yet it is very fast and memory efficient. In terms of retrieval performance, CRT was shown to be very reliable in detecting CRISPRs, based on measures quality, precision and recall. For performance measures tested, CRT outperformed Patscan in all cases. Additionally, when compared to a recently developed CRISPR detection program, Pilercr, CRT showed improved performance under some important conditions. However, using CRT and Pilercr for detecting CRISPRs is recommended, as both are fast and have complementary strengths for precision and recall.

## Availability and requirements

Project name: CRISPR Recognition Tool (CRT)

Project home page: 

Operating system(s): Platform independent

Programming language: Java

## Authors' contributions

CB implemented CRT and wrote the manuscript. NCK and PH guided the research and revised the manuscript. FS, TLR, ML and KDB participated in background research, data collection and tool comparison.
